# Evaluation of Microbiologics CMV verification panel for the quantitation of CMV DNA

**DOI:** 10.1128/jcm.01617-25

**Published:** 2026-03-16

**Authors:** Soya S. Sam, Randall T. Hayden, Sri Suganda, Jessica N. Brazelton, Karen Chandran, Angela M. Caliendo

**Affiliations:** 1 Division of Infectious Diseases, Warren Alpert Medical School of Brown University 12321, Providence, Rhode Island, USA; 2 Department of Pathology, St. Jude Children’s Research Hospital 5417, Memphis, Tennessee, USA; 3 Division of Infectious Diseases, The Miriam Hospital, Brown University Health 6743, Providence, Rhode Island, USA; Wadsworth Center - NYSDOH, Albany, New York, USA

**Keywords:** ddPCR assay, real-time assay, Microbiologics, viral load, quantification, CMV

## LETTER

Quantification of cytomegalovirus (CMV) DNA is essential for the management of transplant recipients and immunocompromised patients. Harmonization of DNAemia results between different test systems and laboratories requires precise, standardized quantitative calibrators (1, 2). While the introduction of World Health Organization (WHO) standards enhanced agreement of quantitative results across tests (3–5), variability persists due to several factors, such as commutability, DNA extraction methodology, target variability, methods used to quantify secondary standards (6, 7), including inconsistent performance of secondary standards with different assays. Here, we evaluated the analytical performance of the Microbiologics CMV verification panel (Microbiologics panel) using multiple commercial automated platforms and external quality control material.

A six-member Microbiologics panel (Microbiologics, St. Cloud, MN, USA), quantified by digital PCR ranging from 7.7 to 2.7 log_10_IU/mL, was prepared from heat-inactivated CMV strain, AD-169 in 10% DNA-depleted human plasma in molecular grade water, supplemented with ProClin 300 and 950 preservatives. Performance of the Microbiologics panel was assessed by testing each concentration from a single lot in triplicate across three non-consecutive days using three real-time PCR assays; RealTi*me* CMV (Abbott Molecular Inc., Des Plaines, IL, USA), Artus CMV RGQ MDx (Qiagen, Germantown, MD, USA), Cobas CMV (Roche Molecular Diagnostics, Pleasanton, CA, USA), and a droplet digital PCR (ddPCR) assay (RealStar ASR CMV reagents [Altona, Plain City, OH, USA]) run on the Bio-Rad QX200 and QXDx ddPCR system (Bio-Rad, Pleasanton, CA, USA). A second lot of the Microbiologics panel was tested in triplicate using RealTi*m*e and ddPCR and included in the data analysis. To determine accuracy, the World Health Organization (WHO) International Standard for CMV (Code: 09/162) (NIBSC, Potters Bar, Hertfordshire, UK, USA) was diluted to 10,000 IU/mL and tested in duplicate on a single run using all assays. Table 1 presents detailed assay information. Linear regression analysis was performed using the GraphPad Prism (ver 10.2.2). Minitab (ver 20.1.1) and Microsoft Excel for Microsoft 365 MSO (Version 2508 Build 16.0.19127.20192) were also used for the analyses.

**TABLE 1:** Characteristics of the assays

Assay	RealTi*m*e CMV (Abbott)	Artus CMV RGQ MDx (Qiagen)	Cobas CMV (Roche)	ddPCR using RealStar ASR CMV Primer (Altona)
Real-time or digital	Real-time	Real-time	Real-time	Digital
Regulatory status	IVD	IVD	IVD	ASR
Extraction method/platform	*m*2000 Sample Preparation System (Abbott)	EZ1 Advanced XL (Qiagen)	cobas 6800 platform (Roche)	EZ1 Advanced XL (Qiagen)
Extraction kit/software	*m*Sample Preparation System DNA	EZ1 DSP Virus Kit	cobas CMV 96 test kit IVD	Virus mini kit v2.0
Cycler/detection system	*m*2000 RealTi*m*e system (Abbott)	Rotor-Gene Q MDx (Qiagen)	Cobas 6800 (Roche)	QX200 and QXDx Droplet Digital PCR system (Bio-Rad)
Target gene	CMV major immediate early gene	CMV major immediate early gene	DNA polymerase (UL54)	
Probe chemistry	Hydrolysis probe	Hydrolysis probe	Probes labeled with target-specific fluorescent reporter dyes	Hydrolysis probe
Amplicon size	105 bp	105 bp	137 bp	
Calibrator type	Plasmid (Exact Diagnostics)	Plasmid (Qiagen)	Internal secondary standard (Roche)	None
Method of calibrator value assignment	Digital PCR	Rotor-Gene Q MDx	Titer based on WHO international standard	

**Fig 1:**
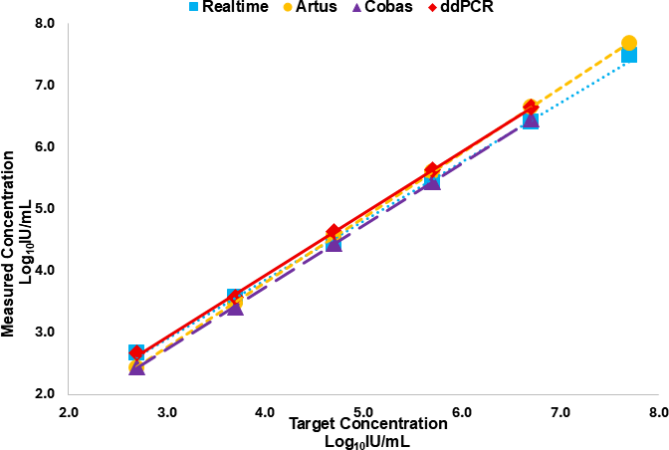
Linear regression analysis of real-time and ddPCR measured values compared against nominal values. *R*^2^ = 0.9932, 0.9995, 0.9993, and 0.9946 for RealTi*m*e, Artus, Cobas, and ddPCR assays, respectively.

There was a strong linear correlation and excellent linearity across the range of the verification panel for all tests (Fig. 1). The concentration of 7.7 log_10_IU/mL was above the quantification limit of both the Cobas and ddPCR assays and therefore was excluded from testing by those systems. Reproducibility was excellent with an overall standard deviation (SD) of 0.07–0.19 (RealTi*m*e), 0.02–0.05 (Artus), 0.02–0.06 (Cobas), and 0.03–0.20 (ddPCR). Bias compared with nominal values of the Microbiologics panel was negative and ranged from 0.03 to 0.29 (RealTi*m*e), 0.02 to 0.28 (Artus), 0.24 to 0.29 (Cobas), and 0.03 to 0.13 (ddPCR) (Table 2; Tables S1 to S4). The WHO standard concentration was comparable across methods: 4.07, 4.04, 3.94, and 3.78 log_10_IU/mL, respectively, for RealTi*m*e, Artus, Cobas, and ddPCR assays.

**TABLE 2:** Comparison of real-time and ddPCR measurements versus nominal values of Microbiologics CMV verification panel

CMV verification panel	Nominal values in log_10_ IU/mL	Mean ± standard deviation (bias) in log_10_ IU/mL			
		RealTi*m*e	Artus	Cobas	ddPCR
Calibrator A	7.70	7.49 ± 0.08 (−0.21)	7.68 ± 0.02 (−0.02)		
Calibrator B	6.70	6.41 ± 0.09 (−0.29)	6.65 ± 0.04 (−0.05)	6.46 ± 0.03 (−0.24)	6.65 ± 0.07 (−0.05)
Calibrator C	5.70	5.42 ± 0.07 (−0.28)	5.61 ± 0.03 (−0.09)	5.44 ± 0.03 (−0.26)	5.64 ± 0.05 (−0.06)
Calibrator D	4.70	4.45 ± 0.13 (−0.25)	4.57 ± 0.04 (−0.13)	4.44 ± 0.02 (−0.26)	4.63 ± 0.03 (−0.07)
Calibrator E	3.70	3.57 ± 0.15 (−0.13)	3.48 ± 0.05 (−0.22)	3.41 ± 0.03 (−0.29)	3.57 ± 0.10 (−0.13)
Calibrator F	2.70	2.67 ± 0.19 (−0.03)	2.42 ± 0.05 (−0.28)	2.45 ± 0.06 (−0.25)	2.67 ± 0.20 (−0.03)

Accuracy of quantitative reference materials is critical for harmonizing results across testing methods. The excellent correlation observed between the Microbiologics panel and assays relative to the nominal values is likely due to normalization to IU and assigned values by ddPCR. Bias was ≤0.29 log_10_ IU/mL with all the assays tested, and accuracy assessed using the WHO standard showed a good agreement between methods. A secondary standard used in viral load determination serves as a reference material to ensure consistency and traceability of quantitative molecular assays. The results of this study indicate that the Microbiologics CMV verification panel is linear, accurate, and reproducible across three commonly used real-time PCR assays.

## SUPPLEMENTAL MATERIAL

Tables S1 to S4 jcm.01617-25-s0001.docx Raw data for all assays.

ASM does not own the copyrights to Supplemental Material that may be linked to, or accessed through, an article. The authors have granted ASM a non-exclusive, world-wide license to publish the Supplemental Material files. Please contact the corresponding author directly for reuse.
