# Supplementary material for: Evaluation of Microbiologics CMV verification panel for the quantitation of CMV DNA
Source: J Clin Microbiol. 2026 Mar 16;64(4):e01617-25. doi: 10.1128/jcm.01617-25 (PMC13059732; doi:10.1128/jcm.01617-25)
Supplement: Tables S1 to S4 — Raw data for all assays. [file jcm.01617-25-s0001.docx]

Table S1: Evaluation of Microbiologics panel using the RealTi*m*e CMV assay

| **Nominal values**  **Log_10_ IU/mL** | **CMV verification panel** | **Lot #1** | | | **Lot #2** | **Mean Log_10_ IU/mL** |
| --- | --- | --- | --- | --- | --- | --- |
|  |  | **Day 1** | **Day 2** | **Day 3** | **Day 4** |  |
|  |  | **Mean Log_10_IU/mL** | | | |  |
| 7.7 | A | 7.48 | 7.54 | 7.53 | 7.4 | 7.49 |
| 6.7 | B | 6.47 | 6.41 | 6.46 | 6.29 | 6.41 |
| 5.7 | C | 5.44 | 5.47 | 5.41 | 5.37 | 5.42 |
| 4.7 | D | 4.58 | 4.44 | 4.51 | 4.27 | 4.45 |
| 3.7 | E | 3.65 | 3.63 | 3.59 | 3.42 | 3.57 |
| 2.7 | F | 2.79 | 2.7 | 2.73 | 2.45 | 2.67 |

Table S2: Evaluation of Microbiologics panel using the Artus CMV RGQ MDx assay

| **Nominal values**  **Log_10_ IU/mL** | **CMV verification panel** | **Lot # 1** | | | **Mean Log_10_IU/mL** |
| --- | --- | --- | --- | --- | --- |
|  |  | **Day 1** | **Day 2** | **Day 3** |  |
|  |  | **Mean Log_10_IU/mL** | | |  |
| 7.7 | A | 7.69 | 7.66 | 7.68 | 7.68 |
| 6.7 | B | 6.67 | 6.62 | 6.66 | 6.65 |
| 5.7 | C | 5.64 | 5.59 | 5.59 | 5.61 |
| 4.7 | D | 4.6 | 4.53 | 4.59 | 4.57 |
| 3.7 | E | 3.5 | 3.45 | 3.49 | 3.48 |
| 2.7 | F | 2.37 | 2.42 | 2.47 | 2.42 |

Table S3: Evaluation of Microbiologics panel using the Cobas CMV assay

| **Nominal values**  **Log_10_ IU/mL** | **CMV verification panel** | **Lot # 1** | | | **Mean Log_10_ IU/mL** |
| --- | --- | --- | --- | --- | --- |
|  |  | **Day 1** | **Day 2** | **Day 3** |  |
|  |  | **Mean Log_10_IU/mL** | | |  |
| 7.7 | A | N/A | N/A | N/A |  |
| 6.7 | B | 6.45 | 6.45 | 6.47 | 6.46 |
| 5.7 | C | 5.40 | 5.46 | 5.45 | 5.44 |
| 4.7 | D | 4.47 | 4.43 | 4.42 | 4.44 |
| 3.7 | E | 3.41 | 3.39 | 3.42 | 3.41 |
| 2.7 | F | 2.45 | 2.46 | 2.43 | 2.45 |

Table S4: Evaluation of Microbiologics panel using the ddPCR assay

| **Nominal values**  **Log_10_ IU/mL** | **CMV verification panel** | **Lot #1** | | | **Lot #2** | **Mean Log_10_ IU/mL** |
| --- | --- | --- | --- | --- | --- | --- |
|  |  | **Day 1** | **Day 2** | **Day 3** | **Day 4** |  |
|  |  | **Mean Log_10_IU/mL** | | | |  |
| 7.7 | A | N/A | N/A | N/A | N/A |  |
| 6.7 | B | 6.61 | 6.63 | 6.59 | 6.76 | 6.65 |
| 5.7 | C | 5.61 | 5.62 | 5.63 | 5.70 | 5.64 |
| 4.7 | D | 4.65 | 4.62 | 4.60 | 4.66 | 4.63 |
| 3.7 | E | 3.60 | 3.53 | 3.58 | 3.58 | 3.57 |
| 2.7 | F | 2.74 | 2.60 | 2.62 | 2.73 | 2.67 |
